# Enhanced learning and retention of medical information in Alzheimer’s disease after differential outcomes training

**DOI:** 10.1371/journal.pone.0231578

**Published:** 2020-04-16

**Authors:** Michael Molina, Isabel Carmona, Luis J. Fuentes, Victoria Plaza, Angeles F. Estévez

**Affiliations:** 1 Escuela de Educación, Facultad de Humanidades, Universidad Mayor, Santiago, Chile; 2 Departamento de Psicología, Universidad de Almería, Almería, Spain; 3 Departamento de Psicología Básica y Metodología, Universidad de Murcia, Murcia, Spain; 4 Departamento de Psicología Básica, Universidad Autónoma de Madrid, Madrid, Spain; 5 Departamento de Psicología, Universidad Autónoma de Chile, Santiago, Chile; 6 CERNEP Research Center, Universidad de Almería, Almería, Spain; Nathan S Kline Institute, UNITED STATES

## Abstract

**Background:**

Adherence to treatment is a crucial factor for patients who have chronic illnesses or multiple morbidities and polypharmacy, which is frequently found in older adults. The non-adherence to medications has important economic and social consequences as well as impacts on the health of the patients. One of the reasons that can explain the low adherence to treatment, is the memory deficits that are characteristics of this population and that are even more evident in cases that involve neurodegenerative diseases.

**Methods and findings:**

In this study, we explore whether the differential outcomes procedure (DOP), which has been shown to be useful in improving discriminative learning and memory in different populations, may facilitate learning and retention of medical recommendations in older adults who have been diagnosed with Alzheimer’s disease. The results demonstrate that when this procedure was applied, the patients showed improvements in learning and long-term retention of two pill/time of day associations in a situation that simulates adherence to medical prescriptions.

**Conclusions:**

These findings contribute new data about the potential benefits of the DOP in patients with neurodegenerative disorders, highlighting the important role that this procedure could play in addressing important issues related to the health and quality of life of older adults, with or without neurodegenerative diseases, such as low adherence to medical treatments.

## Introduction

It is well known that medicine has grown exponentially over recent decades. The advances in the different disciplines that converge within medicine have not only made it possible to have timely and precise diagnoses of different pathologies but also resulted in the development of treatments for practically all diseases or, failing this, the possibility of treating symptoms, boosting the quality of life of those who are afflicted by these diseases. However, we are faced with a challenge that for many years has attracted the attention of governments, public health institutions, and international organizations that have expressed their concerns over patients’ lack of adherence to medical treatment [1].

In 2003, the World Health Organization [2] defined adherence to treatment as the degree to which the drug-taking behavior, follow through on a diet, or modification of living habits of a patient corresponds to the recommendations of healthcare professionals. In that report, it is also indicated that approximately 50% of patients have difficulties in following their medical prescriptions (they do not take the medication as it has been prescribed), which lead to unnecessary prolongation of diseases or to death. It is worth noting that non-adherence to medical prescriptions still remains as a critical public health issue [3]. In fact, different studies have observed a direct relationship between the lack of adherence to treatment of patients and the increment in the budget of local public health systems [1,4–6]. Furthermore, Silva, Galeano, and Correa [7] indicate that adherence-to-treatment issues are present in all types of pathologies, among all age groups, in different life stages, and, in particular, in patients who have chronic treatments, which affect an important part of the population.

Some authors have noted that ageing is one of the most important factors related to adherence to treatment shown by patients (e.g., [8,9]). Thus, Leal, Abellán, Casa, and Martínez [10] observed that adherence to medication decreases as the number of pills that patients must take every day increases, mainly for patients older than 65 years with chronic illnesses. In other words, the greater the number of medications is, the lower the percentage of fulfilling the medical recommendations will be. Specifically, it is expected that 75% of people older than 65 years will correctly follow the treatment when they must take one pill; 68% when the medical prescription is two; 54% when three pills are prescribed; and 35% when the doctor prescribed four pills. This trend continues to progressively decrease until it reaches 10% adherence for those who must take nine medications a day [10]. This polymedication condition, that involves the management of complex medication regimens, places an excessive burden on memory, highlighting one of the most frequent issues associated with age-related cognitive deterioration, a deficit in working memory capacity (e.g., [11,12]). In fact, it is known that approximately 80% of the information provided by doctors to patients will be forgotten immediately or remembered incorrectly, particularly when they are older or they are anxious, making patients’ forgetfulness of taking their prescribed medication one of the main issues for adherence to treatment [13,14]. Note that explicit memory deficits of prescribed information dramatically increase when patients present any neurodegenerative disease, especially when dementia is involved [15], or multiple morbidities that require several medications daily. These conditions increase the burden on mnemonic resources, which could explain the low treatment adherence rate that characterizes this population (e.g., [16]). In accordance with this, it has been found that the higher the memory scores from the memory subscale of the Dementia Rating Scale (DRS, [17]), the better the adherence to treatment observed in older adults with cognitive impairment [18]. Considering the important economic and health consequences of low adherence to medical prescriptions, developing new proposals that facilitate adherence to medical treatment, particularly in cases of older adults who have multiple chronic diseases, would be very welcome. Such kinds of proposals should be designed to foster memory of medical prescriptions and the active participation of the patients, for instance, through the use of mnemonic techniques that may facilitate learning schedules, the dosage, or the identification of medicines. In the present study, we aim to assess the benefits of one of these techniques, known as the differential outcomes procedure ([19]), in the context of adherence to treatment in dementia.

**Fig 1:**
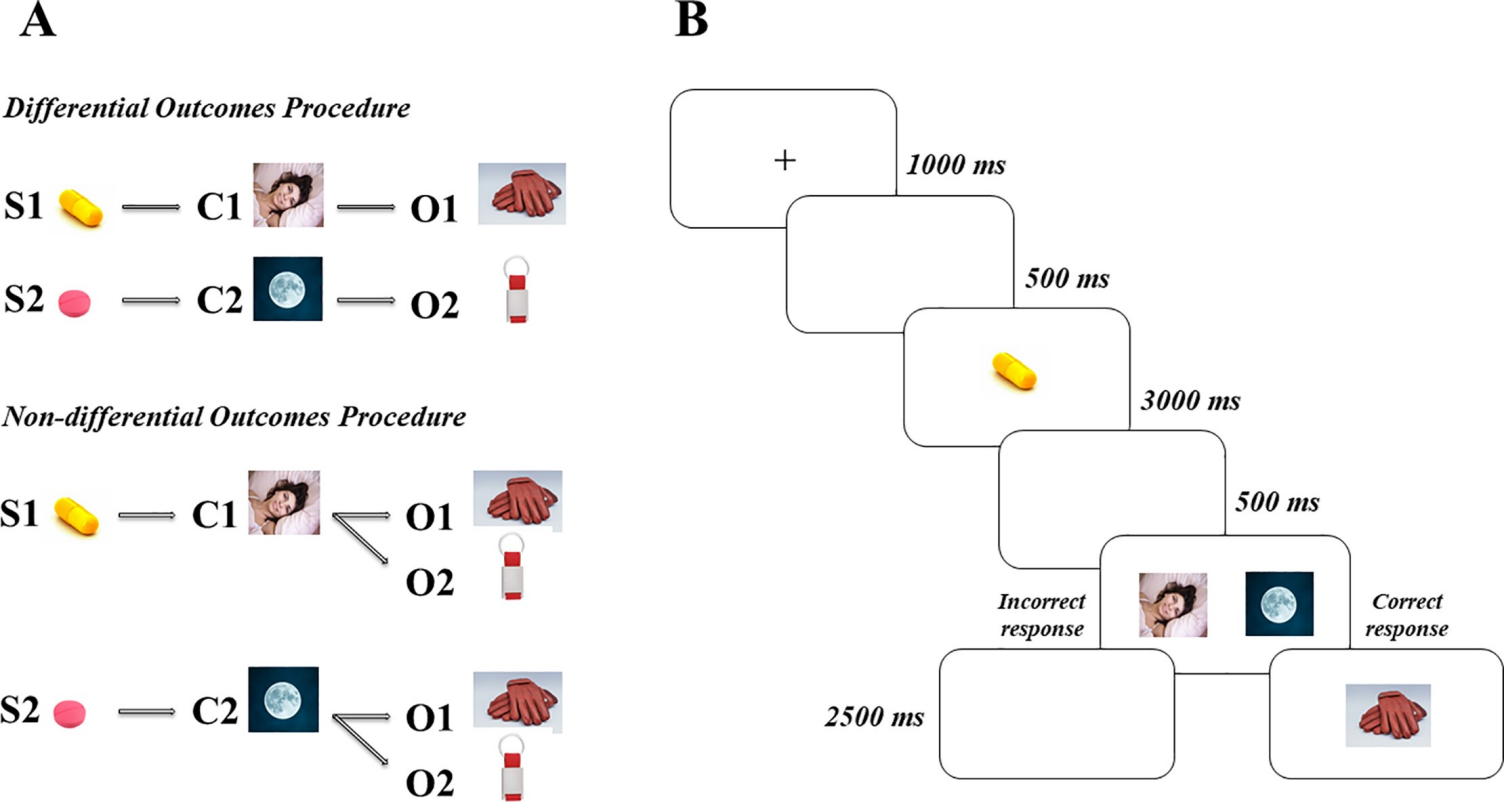
A) Illustration of both differential (above) and non-differential (below) outcomes procedures. B) Stimulus sequence (from left to right) used in the learning task. S = sample stimulus; C = comparison stimulus or choice; O = outcome.

The use of outcomes-based procedures stems from the animal experimental tradition in which the outcomes (reinforcers) after correct responses are administered to improve discrimination learning. In discrimination learning, the participant (animal or human) is presented with a set of stimuli (for instance, a plate with soup or a plate with pasta), which are referred to as the *sample stimuli*. Then, a set of choices is displayed (for instance, a spoon and a fork), which are referred to as the *comparison stimuli*. Participants have to learn, in a trail-by-error manner, which sample stimulus goes with each comparison stimulus. A *delayed-matching-to-sample* task is used if there is a temporal interval between the presentation of one of the sample stimuli and the presentation of the comparison stimuli (choices) in each trial. When the choice (response) is correct, an outcome (reinforcer) comes up to provide the participant with feedback about the correctness of its/his/her responses. Usually, all outcomes are provided interchangeably in a random manner when the more standard non-differential outcomes procedure (hereafter the NOP) is used. However, what characterizes the differential outcomes procedure (hereafter the DOP) is that each outcome is unique to each sample stimulus-comparison stimulus association. For instance, in the presence of a plate of soup, choosing the spoon may always be reinforced with the outcome “well done”, whereas in the presence of a plate of pasta, choosing the fork may always be reinforced with the outcome “correct” (see Fig 1A for an illustration of the two outcomes procedures). The DOP has proven its effectiveness in improving discriminative learning in animals (see [20], for a review), children (e.g., [21]), university students (e.g., [22]), and patients (e.g., Down syndrome, [23]), and recent studies have extended its application to populations with recognition memory deficits (for a systematic review, see [24]).

In terms of the therapeutic utility of the DOP, recent research has addressed daily deficits shown by patients with memory recognition deficits. For instance, Hochhalter, Sweeney, Bakke, Holub, and Overmier [25] found that four patients with alcohol dementia, who often had difficulties with delayed facial recognition, improved recognition of familiar faces when each face was associated to a specific outcome (DOP) in comparison to when outcomes were randomly administered (NOP). An advantage of the DOP compared to the NOP was also observed by López-Crespo, Plaza, Fuentes, and Estévez [26] with older adults who displayed the typical memory deterioration associated with ageing. Also, Plaza, López-Crespo, Antúnez, Fuentes, and Estévez [27] carried out a study with eight older adults with Alzheimer’s disease (AD) to assess whether the use of the DOP would have an impact on recognition memory performance in these patients, promoting some improvement in delayed facial recognition. The results indicated that patients with AD had a higher accuracy in the memory tests when the outcomes (photographs of an umbrella, a scarf, a perfume bottle, and a mug, which were raffled off at the end of the study), were administered in a differential manner (the DOP), in comparison to when the outcomes were administered in a random manner (the NOP). These findings demonstrated, for the first time, that patients with this type of dementia could benefit from the application of such a simple training procedure to ameliorate their deficits in recognition memory. Similar results have been found in two recent studies indicating the effectiveness of the DOP in improving visual recognition memory for non-facial visual stimuli [28] as well as spatial working memory [29] in this population.

Of special interest for the objectives of the present research is the study of Molina, Plaza, Fuentes, and Estévez [30] with healthy young adults. The authors used a great number of illness-treatment pairings to simulate daily conditions of many older adults, with or without age-relate neurodegenerative diseases, who have to learn which medicines are appropriate for their multiple diseases. The results indicated that, compared to the standard or non-differential outcomes procedure (the NOP), when each to-be-learned association was reinforced with a unique outcome (the DOP), participants showed higher percentage of correct responses, needed fewer trials to learn the associations, and demonstrated greater long-term retention of the information that was previously learned. In a recent follow-up study, Plaza, Molina, Fuentes, and Estévez [31] demonstrated that healthy older adults also benefited from the DOP when the to-be-learned association was between a particular pill and the time of the day the participants had to take it according to a prescribed schedule. The benefits of the DOP extended also to performance in a recognition memory test that took place one week later. The results showed the expected greater learning and long-term retention when the DOP was used compared to the NOP. These findings suggest that the DOP could be deemed as an appropriate complementary strategy in intervention programs that aim to increase adherence to medical treatments, particularly in people with learning and memory deficits that have serious impact on their everyday lives.

In the present study, we sought to assess the benefits of the DOP in patients who have been diagnosed with AD, in conditions that simulate adherence to medical treatment. Concretely, we asked whether the implementation of the DOP with these patients would benefit their ability to learn and remember the time-based schedule of the medication administration as might had been prescribed by a doctor, which could have a beneficial impact on their adherence to treatment and therefore on their health. To that end, we used a task that simulated the adherence to a new pharmaceutical treatment in which patients had to learn to associate two medicines (two pills) with the time of day (morning and night) they should be taken (see [31]). Outcomes consisted of two reinforcers (e.g., a pair of gloves and a keychain) that could be administered under the DOP (a unique outcome followed each determined pill/time of day association) or under the NOP (each outcome could be administered after each pill/time of the day association in a random manner). A repeated measures design was employed in this study. Consequently, all participants carried out the learning task and the memory tests under both outcomes conditions in a counterbalanced way. Memory tests were conducted 1 hour and 1 week after the learning training sessions. On the basis of the results observed by Molina et al. [30] and Plaza et al. [31], as well as those of other studies that have shown long-term memory improvements under the DOP (e.g., [28,32,33]), we hypothesized that patients with AD will show improved learning and long-term retention of the learned associations when they are trained with the DOP compared to when they are trained with the NOP.

## Method

### Participants

Twenty-two participants, eleven healthy controls (HC) and eleven patients with Alzheimer’s disease, participated in the study. A priori power analysis was performed with the G*Power software 3.1.9.2 [34] in order to determine the minimum required sample size to detect main effects and interactions. With an alpha = .05 and power = .80, the analysis showed that twelve participants were required to detect a medium effect size (d = 0.64) according to previous studies that used the DOP with AD patients [27–29].

AD patients were recruited from Virgen de la Esperanza and La Purísima senior assisted living homes (Almería, Spain). An experienced neurologist established the diagnosis on the basis of the National Institute of Neurological and Communicative Disorders and Stroke (NINCDS), and Alzheimer’s Disease and Related Disorders Association (ADRDA) criteria for probable Alzheimer's disease [35,36]. Only patients in phase 4 of the Reisberg’s global deterioration scale [37] participated in the study. They also had a relatively low MMSE score indicating moderate cognitive impairment (mean MMSE = 19.6). Exclusion criteria included (i) any neurological disease other than AD such as frontal lobe dementia, vascular dementia, alcohol dementia, Lewy body dementia, normal pressure hydrocephalus, brain tumor, Parkinson’s disease, or Huntington’s disease, and (ii) health conditions or medications that prevented patients from joining the study. The HC participants were recruited from the community and were free from serious medical conditions (i.e., heart disease, cancer, neurological disease including dementia, psychiatric illness, substance abuse disorder, or substance dependence). They all scored above 24 in the MMSE. Table 1 shows demographic and clinical information for participants of the two groups (AD and HC). This study was approved by the University of Almería Bioethics Committee in Human Research, and it was conducted according to the ethical protocols and recommendations of the ‘Code of Good Practices in Research’ of this committee and to the principles expressed in the Declaration of Helsinki. Informed written consent was obtained from all participants or patient’s caregiver. Finally, two AD patients and one participant from the HC group did not complete the training (they all missed one session), and consequently the final sample included 10 HC participants and 9 AD patients.

**Table 1:** Demographics and clinical information for the HC and AD groups. Mean scores and standard deviations (in parentheses) are shown.

*Socio-demographic data and tests*	*Maximum*	*HC*	*AD*
*n*		10	9
Sex (F/M)		6/4	6/3
Age		74(10.3)	73.7(9.6)
MMSE**	30	28.7(1.6)	19.6(4.7)
GDS**	7	1.5(0.5)	4(0)
CERAD Battery			
Boston Naming Test**	15	14.4(1.1)	6.7(3)
Word List Memory**	10	6.3(2.3)	2.2(1.8)
Word List Recall**	10	5.5(2.8)	0.9(0.8)
Word List Recognition**	20	17.3(4.5)	9.4(5.8)
Constructional Praxis**	11	9.1(3.5)	0(0)
Trail Making Test (part A)**		61.3(43.4)	136(18.5)
Trail Making Test (errors)**		0(0)	1.3(1.1)
Barcelona Test (Subtests)			
Constructive Praxis to the Copy**	18	17.1(1.5)	4.7(3.3)
Semantic Fluency**		18.5(5.3)	8.7(3.1)
Phonological Fluency (P) **		19.7(10.3)	6.9(3.2)
Forward Digits Span	9	5.5(1.4)	4.1(1.1)
Backward Digits Span**	8	3.3(0.8)	1.7(1.1)
Abstraction**	12	9.4(3.1)	3.4(2.1)
Reciprocal Coordination**		14(2.1)	1.9(1.2)
Beats count		8.2(4.2)	9(5.8)

MMSE = Mini-Mental State Examination; GDS = Global Deterioration Scale; CERAD = The Consortium to Establish a Registry for Alzheimer’s disease; HC = healthy controls; AD = Alzheimer’s disease.

**p<0.01

### Stimuli and materials

The sample stimuli consisted of two sets of pictures of two different pills (set one: a yellow pill and a pink pill; set two: a blue and pink pill and a blue and red pill). The comparison (choice) stimuli consisted of two pictures representing two times of day (morning and night). The outcomes (reinforcers) consisted of two sets of pictures representing the gifts that the participants could receive at the end of the study an served to provide feedback about whether the response (choice) was correct or not (set one: a pair of gloves and a keychain; set two: a pair of socks and a mug). All stimuli were displayed covering an area of 5.5 X 6.5 cm. E-Prime 2.0 software [38] controlled stimulus presentation as well as participants’ responses (accuracy and latency data). All stimuli were presented with a white background on a color touch TFT-LCD computer screen. The reinforcers were raffled off at the end of the experiment. All participants received one of them and an acknowledgment diploma for their participation.

### Procedure

**Fig 2:**
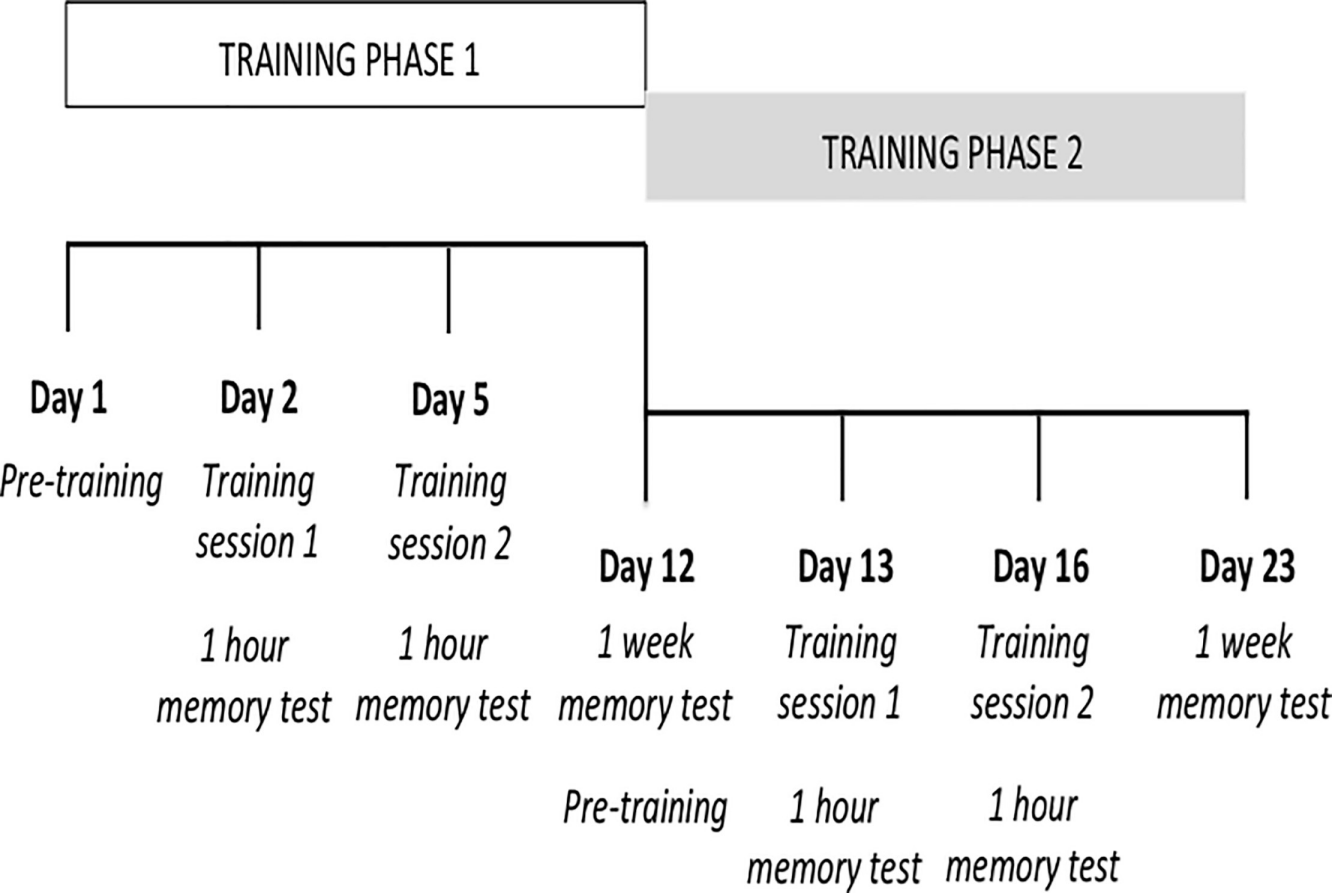
**Sequential progression (from left to right) of the two phases of the study.** The memory task was performed one hour after each of the two training sessions and a week after the last training session of each phase (see Fig 2). The memory task consisted of two trials, one with each trained pill-time of day association, identical to that used during the training sessions except that outcomes were not administered at all. That is, as it is depicted in Fig 1B, participants first saw the picture of a pill and, after a short delay (500 ms), they had to choose, by touching the screen, the picture of the time of day associated with the pill. After a response was made, the screen remained blank for 2500 ms, and then the next trial began.

All participants were tested individually in a quiet room. We used a delayed-matching-to-sample task that consisted of two phases (1 and 2), separated by one week. Each phase consisted of a pre-training session followed one day later by training session 1 and then two days later by training session 2 (see Fig 2). Each training session lasted approximately 30 minutes. At the beginning of each session, participants were instructed that the task required to guess first, and remember later on, which time of day was associated with the pill they had just been presented with, according to the supposed medical treatment prescribed by a doctor. Instructions were verbally given by the experimenter while a sample trial was shown on the screen. Then, they performed one practice trial (pre-training session) or four practice trials (training sessions) before completing 36 training trials grouped in three blocks of 12 trials each. The order of the blocks was counterbalanced across participants.

Each trial began with a central fixation point (#) during 1000 ms followed by a blank screen for 500 ms. Then, one of the sample stimuli (the picture of a pill) appeared in the middle of the screen for 3000 ms. After a 500 ms delay interval with a blank screen, the two comparison stimuli (the morning and night pictures) were presented until the participants responded. Participants were asked to touch the picture of the time of day that was associated with the sample pill they had just been presented with. Responses were followed by either the picture of a reinforcer (the outcome) when they were correct, or a blank screen when they were incorrect, in both cases during 2500 ms. Then, the experimenter pressed the space bar and the next trial began. Fig 1B illustrates the sequence of events of one trial.

As mentioned above, the pre-training session was administered to all participants to ensure that they understood the task demands before the two training sessions started. At the beginning of phase 1 session, and before the training sessions started, participants were randomly assigned to one of the two outcomes conditions, differential (DOP) or non-differential (NOP), according to a computer-generated blocked randomization schedule. Participants that during phase 1 were assigned to the DOP, during phase 2 performed the training sessions under the NOP, and vice versa. Under the DOP, each pill/time of day association was consistently reinforced with a determined outcome when the participant’s choice was correct (yellow pill/morning association could be reinforced with the outcome “a pair of gloves”, whereas pink pill/night association could be reinforced with the outcome “a keychain”). Under the NOP, each pill/time of day association was reinforced with one of the outcomes in each trial, so that the two outcomes followed each pill/time of day association an equal number of times across the training session. A week later, participants were trained with the same task during another three sessions (phase 2) but using the other set of two pill-time of day associations (e.g., the blue and pink pill/morning and the blue and red pill/night associations) under the opposite outcomes procedure condition.

### Statistical analysis

Percentages of correct responses for each participant were grouped in three blocks of 12 trials in each training session. Sex did not produce any statistically significant effect nor interacted with any other factor, and consequently data from women and men were collapsed. Data were submitted to a mixed ANOVA with Group (AD and HC) as the between-participants factor and Outcomes (DOP and NOP), Training Session (1 and 2) and Blocks (B1, B2 and B3) as the within-participants factors. Latency data did not show any significant effect and therefore are not reported.

Because the pattern of results was similar for the memory task performed one hour after each of the two training sessions (a short retention interval), data from these two memory tasks were also collapsed for the statistical analyses. Percentages of correct responses from the short- (one hour) and long-term (a week) memory tests were finally submitted to a mixed ANOVA, with Group (AD and HC) as the between-participants factor and Outcomes (DOP and NOP) and Retention interval (short and long) as the within-participants factors. One AD patient and one HC participant did not complete one of the tests in one of the Outcomes conditions–DOP or NOP–so their data were excluded from the memory tests analysis. The statistical significance level was set at p ≤ .05.

## Results

### Training sessions

**Fig 3:**
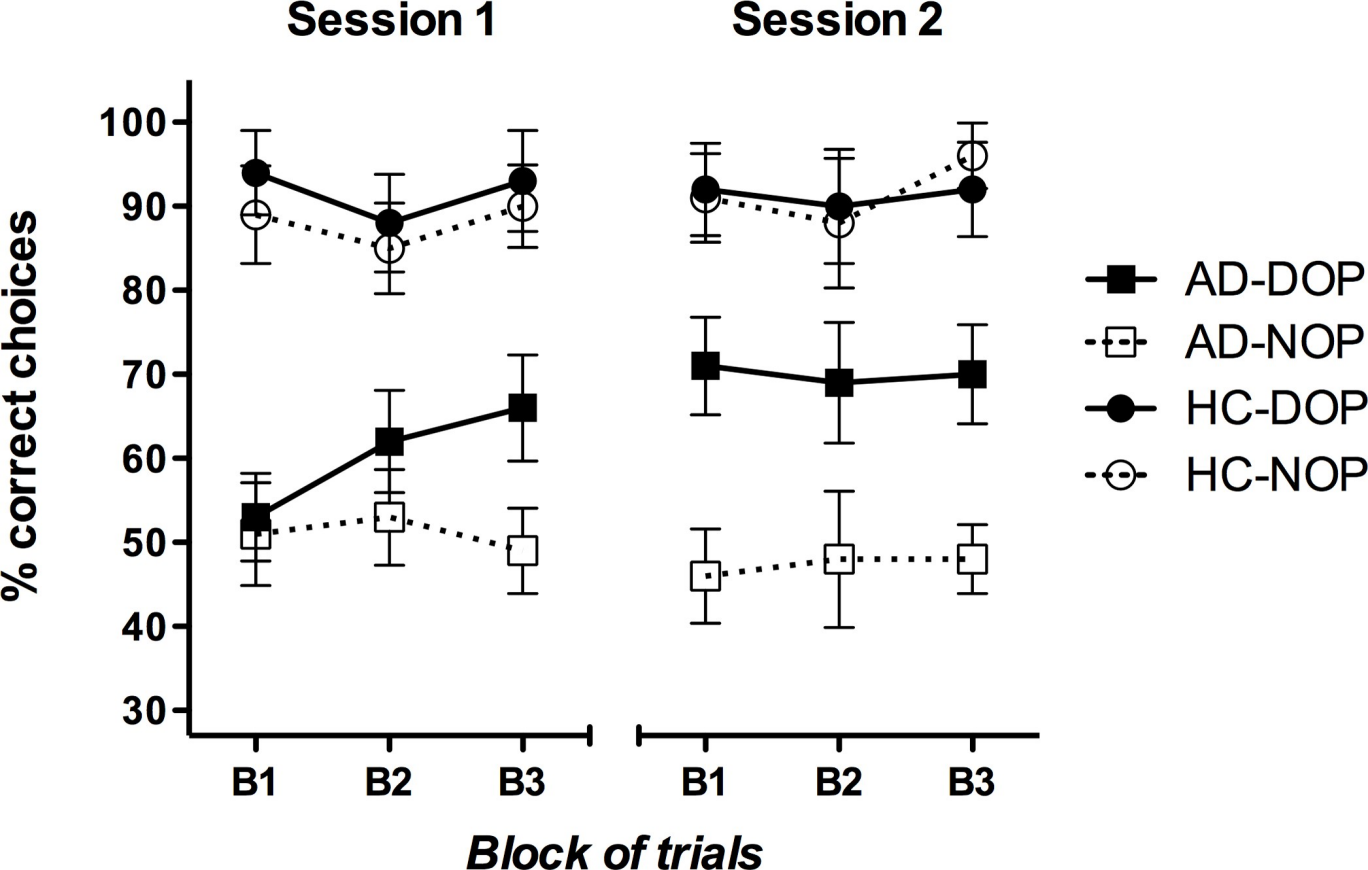
Mean percentages of correct responses obtained by participants in the discriminative leaning task as a function of the training session (1 and 2), blocks of trials (three blocks of 12 trials each), outcomes (DOP and NOP) and group (patients with Alzheimer’s disease–AD- and healthy controls–HC-). Error bars show the standard error of the mean.

Results from the accuracy data analysis showed significant main effects of Outcomes [*F*(1,17) = 8.79, *p* = 0.009, η_p_^2^ = 0.341] and Group [*F*(1,17) = 26.93, *p* < 0.001, η_p_^2^ = 0.613]. Healthy controls were more accurate than the patients (91% vs. 57% correct, respectively); and participants performed the task better in the DOP than in the NOP condition (78% vs. 69% correct, respectively). The two-ways Outcomes X Group interaction was significant [*F*(1,17) = 5.60, *p* = 0.030, η_p_^2^ = 0.248]. The interaction showed that the DOP produced significantly higher accuracy than the NOP just in the AD group [*F*(1,8) = 6.91, *p* = 0.030, η_p_^2^ = 0.463], but not in the HC group [*F*(1,9) = 1.26, *p* = 0.290, η_p_^2^ = 0.123]. Importantly, the Outcomes X Training Session X Group interaction was also significant [*F*(1,17) = 9.05, *p* = 0.008, η_p_^2^ = 0.347]. The three-ways interaction analyses revealed that the Outcomes X Training Session partial interaction was significant just for the AD group [*F*(1,8) = 17.90, *p* = 0.003, η_p_^2^ = 0.691], but not for the HC group [*F*(1,9) = 0.74, *p* = 0.412, η_p_^2^ = 0.076]. Whereas performance in the DOP and the NOP was equivalent across the two sessions in the HC group, in the AD group an Outcomes effect was observed in the second session [*F*(1,8) = 10.53, *p* = 0.012, η_p_^2^ = 0.568]. That is, the beneficial effect of the DOP in AD patients’ discriminative learning was evident only in the last training session (70% vs. 47% correct for DOP and NOP, respectively). To further explore this learning effect, the six blocks of trials (three from each session) were analyzed for each group in each Outcomes condition separately. It is worth noting that only in the DOP condition patients’ performance showed a significant learning curve [Blocks effect: *F*(5,40) = 4.03, *p* = 0.005, η_p_^2^ = 0.335]. As it can be observed in Fig 3, AD patients did not learn the task when trained with the NOP (their performance was at chance in all blocks).

### Memory tests

**Fig 4:**
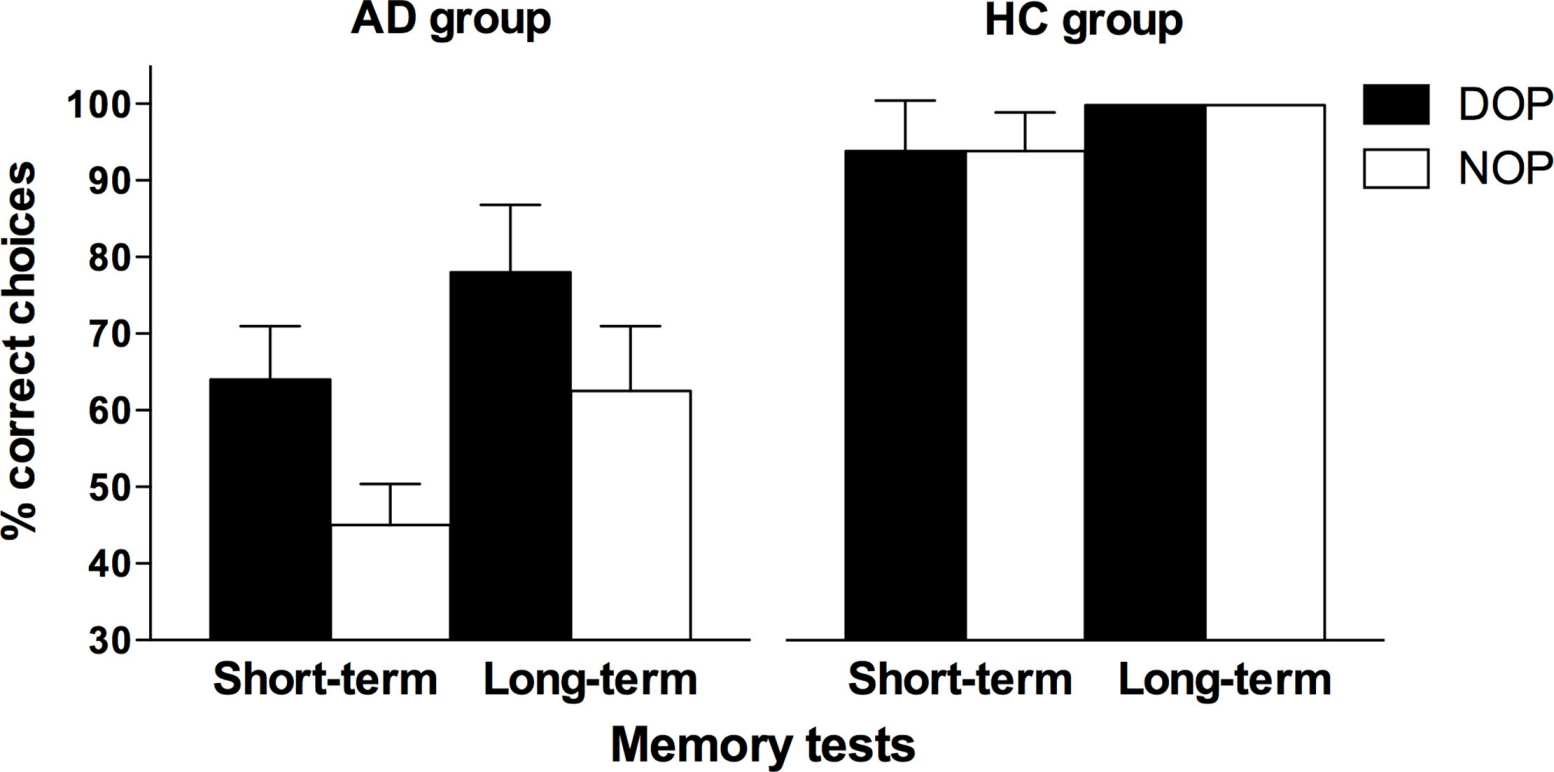
Mean percentages of correct responses obtained by participants in the memory tests as a function of retention interval (short and long), outcomes (DOP and NOP) and group (patients with Alzheimer’s disease–AD- and healthy controls–HC–). Error bars show the standard error of the mean.

Fig 4 shows the percentage of correct responses in the memory tests. The results revealed significant main effects of Group [*F*(1,15) = 40.24, *p* < 0.001, η_p_^2^ = 0.728] and Outcomes [*F*(1,15) = 6.91, *p* = 0.019, η_p_^2^ = 0.315]. The AD patients’ performance in the memory tests was lower than that of the HC participants (63% vs. 97% correct), and participants showed an overall better retention of the learned associations when they were trained with the DOP (84% correct) than when they were trained with the NOP (76% correct). The two-ways Outcomes X Group interaction reached statistical significance [*F*(1,15) = 6.91, *p* = 0.019, η_p_^2^ = 0.315]. The analysis of the interaction revealed a significant effect of Outcomes in the AD group [*F*(1,7) = 6.89, *p* = 0.034, η_p_^2^ = 0.496] but not in the HC group [*F*(1,8) = 0.00, *p* = 1.000, η_p_^2^ = 0.000]. That is, only AD patients showed an improvement in memory performance when the DOP and the NOP conditions were compared (71% vs. 54%, respectively), being their performance at the chance level just in the NOP condition.

## General discussion

It is expected that a pharmaceutical treatment will meet its goal when there is at least an 80% adherence to treatment (e.g., [1,39]). Nevertheless, this percentage could be impacted by several variables such as the severity of the disease, cognitive deterioration, age, errors in dosage, forgetting to take medications, or mistaking medications, which impact not only the fulfillment of medical advice but also the quality of life of the patients (e.g., [40,41]). Consequently, the development of new therapeutic strategies that may facilitate adherence to treatment, particularly in the more advanced stages of life when cognitive problems (e.g., memory loss) and health issues increase, should be deemed of crucial relevance. The present study was conducted with the main aim of assessing whether one of these possible strategies based on learning through differential outcomes, would facilitate the learning and retention of medical prescriptions in adults diagnosed with AD, in a simulated medical treatment context. Accordingly, our AD patients learned the pill/time of day association task when they were trained under the DOP, but not when they were trained under the NOP, performance being at chance level just in the latter condition. A detailed inspection to the present findings in comparison with those of previous related studies suggest that the amount of training is an important factor for the benefits of the DOP to be observed with these kinds of patients. For instance, whereas Plaza et al. [31] observed DOP benefits when healthy older adults received just one single training session, in a task similar to the one employed in the present study, AD patients in the Vivas et al. [29] study showed moderate improvements in spatial recognition memory with just one training session, and benefits restricted just to the last block of trials. It led us to increase the number of training trials here, as we anticipated that our AD patients would exhibit greater difficulty in learning/remembering the pill/time of day associations, compared with healthy adult participants. Note also that it is likely that learning tasks like the one used in the present study require the activation of two processes that were deteriorated in the AD population: discrimination learning, involved in the establishment of the pill-time of day associations, and short-term memory, given that a delay of 500 ms was interposed between both types of stimuli, sample stimuli and comparison (choice) stimuli.

Regarding long-term memory of the learned associations, we also found an overall advantage when the DOP methodology was used during the training sessions. The effect was particularly important for patients with AD who, again, showed a performance close to chance with the NOP (54% correct), which increased considerably when they were trained with the DOP (71% correct). These results are relevant because, for the first time, reveal an improvement in long-term retention of information previously learned by using the DOP, in people who have been diagnosed with a neurodegenerative disease usually associated with learning and memory deficits (e.g., [42]). This finding adds to that reported by Carmona et al. [28] using a visual recognition task. Specifically, the memory decline (less hits, more false alarms, and a lower discriminability) that was observed in eight AD patients at the 1-week memory test, was not found when the patients were trained with the DOP. Taken together, the results from both studies highlight the potential of the DOP to improve long-term retention in people with memory impairments.

Finally, in contrast to what Plaza et al. [31] observed with older adults, the results of our control group (healthy older adults) did not reveal significant differences irrespective of the learning outcomes condition to which the participants were assigned. In other words, their performance oscillated between 90% and 100% accuracy, regardless of how the reinforcers were administered after their correct responses. These results suggest a ceiling effect in this group of participants who found the task very easy to learn. In fact, previous studies have shown that when the difficulty of the task is low, the DOP does not produce any benefit or improvement in either latency or accuracy data (e.g., [21,22,43]). However, it should be noted that when the demands of the task increased (by increasing, for example, the number of pill/time of day associations to three, see [31]), the use of the DOP positively affected both discriminative learning and long-term retention of information in older adults without cognitive impairments. Therefore, taken together, all these studies suggest that learning under the DOP can be deemed as a useful and complementary strategy to increase adherence to treatment in normal and pathological aging.

A final question refers to the mechanisms by which the DOP exerts its beneficial effects on performance. The two-memory systems model [44], an evolution of the Expectancy Theory [45], is currently the most comprehensive account of how the DOP works. In short, primarily based on studies conducted with animals, Savage and colleagues (e.g., [46–49]) suggested the existence of two well-differentiated neurochemical and neuroanatomical systems that become activated independently, depending on how the reinforcers are administered, either differentially as in the DOP, or non-differentially as in the NOP. Specifically, when the outcomes are presented randomly, or non-differentially, the explicit retrospective memory system is activated. This system is dependent on the cholinergic system and is linked to the normal functioning of the hippocampus. On the other hand, when the administration of outcomes is specific, as in the DOP, an implicit prospective memory system is activated, a type of memory related to the glutamatergic system that involves the basolateral amygdala in animals (see [49], for a review). Prospective memory of the unique outcome associated with each sample stimulus would guide the selection of the appropriate choice. Thus, the selection of the correct comparison stimulus will not depend exclusively on the explicit retrospective recall of the sample stimulus.

Several studies conducted with humans have supported this model, particularly the study by Mok, Thomas, Lungu and Overmier [50], who investigated the brain structures that are linked to the functioning of the DOP in humans. In this study, brain activity was explored through functional magnetic resonance imaging (fMRI) while the participants performed a discriminative learning task that was reinforced under the DOP or the NOP, with auditory (a non-vocal initial excerpt from the pop song Macarena), or visual (three smiling baby pictures presented successively) outcomes. Mok et al. [50] found that activation of the hippocampus increased during the delay interval between the sample stimulus and the comparison stimuli when the participants were performing the task under the NOP, which indicate that this structure is involved in the retrospective processing of information in humans too. However, unlike the results obtained with animals, when the participants were trained with the DOP, increased activation was observed in brain areas related to the sensory nature of the outcomes. Thus, when the discriminative stimulus was associated to a visual outcome (smiling baby pictures), the areas involved in the processing of visual information (i.e., Brodmann areas 18 and 19) were activated during the delay. On the other hand, when the associated outcome was an auditory stimulus (a non-vocal excerpt from the song Macarena), the areas involved in the processing of this type of information (i.e., Brodmann areas 41 and 22) were activated. Furthermore, in both cases, the activation of the angular gyrus of the posterior parietal cortex was observed, indicating its involvement in prospective memory. A recent study has also demonstrated that, as the two-memory system model suggests, this prospective memory is largely implicit [51]. In fact, participants showed a better visual recognition memory when the DOP was applied regardless of whether the outcomes or the sample stimuli were presented under subliminal (non-conscious) or supraliminal (conscious) conditions.

## Limitations and conclusions

A first limitation of the present study is the small sample of participants in both groups. Although further studies with ample samples are always an important research goal, it is worth noting that we have observed a clear-cut pattern of results that systematically replicates a main finding: the benefit of using differential outcomes when people with or without neuropathology have to learn and retain in memory determined stimulus-response associations. A second limitation of the study is that the task might have been very easy for the control group, who learned it without great effort, showing ceiling effects. However, the main aim of this particular study was not to explore DOP benefits in our adherence to treatment simulation in the elderly, but whether such procedure may be of any help to ameliorate the learning/memory deficits in AD patients, with a task that is appropriate to this kind of population. A third limitation is that sociodemographic as well as affective states of the participants may play a role in the efficiency with which people perform learning and memory tasks. Further research should explore how these factors interact with the learning procedure to determine how well patients adhere to medical prescriptions.

To sum up, the results obtained in the present study, along with those from previous studies [25,27–29] suggest that the use of the DOP could be very useful in training patients with learning and memory deficits that result from damage to the hippocampus or the dopamine system. Such is the case of the target population of this study, older adults who have been diagnosed with AD. Importantly, our findings indicate also that this procedure could be integrated into more comprehensive training programs with the goal of improving the well-being and the quality of life of these patients as well as of older adults with or without neurodegenerative disorders. One example of this sort of programs is the promotion of adherence-to-treatment behavior through the learning and retention of the drugs patients must take at specific times throughout the day. Finally, it is worth noting that the DOP is an inexpensive method that simply consists in consistently associate each correct stimulus-choice sequence with a unique outcome (for example, a specific reinforcer). It makes the procedure easy to be implemented not only by healthcare professionals but also by family members or caretakers, which widens its scope of action and increases the possible benefits that this procedure may have for the physical and psychological health of different populations.
